# Assessing male involvement in childcare and associated factors among fathers of below two in Toke Kutaye district, Central Ethiopia, 2024: a community-based cross-sectional study

**DOI:** 10.3389/fpubh.2025.1527675

**Published:** 2025-03-12

**Authors:** Gemechu Ganfure, Jiregna Darega, Latera Debebe Kitila

**Affiliations:** ^1^Department of Pediatrics and Child Health Nursing, Ambo University College of Medicine and Health Science, Ambo, Ethiopia; ^2^Department of Public Health, Ambo University College of Medicine and Health Science, Ambo, Ethiopia; ^3^Department Toke Kutaye District Health, Oromia Regional Health Bureau, Oromia, Ethiopia

**Keywords:** male involvement, child care, Toke Kutaye, Ethiopia, fathers

## Abstract

**Background:**

Male involvement in child care is increasingly acknowledged as a crucial factor for promoting positive health and developmental outcomes for children, particularly during the critical early years of life. In Ethiopia, low male participation in child-rearing exacerbates public health issues, including high neonatal and under-five mortality rates. This study examines the prevalence and factors associated with paternal involvement in child care among fathers of children under 2 years in Toke Kutaye District, Central Ethiopia.

**Methods:**

A community-based cross-sectional study was conducted from January 28 to February 16, 2024. A total of 605 fathers with children under 2 years were randomly selected for interviews. Data on male involvement in child care were collected using a structured questionnaire. Data were entered using Epi-data version 3.1 and analyzed using SPSS version 26. Bi-variable and multivariable logistic regression analyses were performed, applying adjusted odds ratios (AOR) with 95% confidence intervals (CI) to assess statistical significance, with a *p* < 0.05. Descriptive statistics were also computed and presented using text, charts, and tables.

**Results:**

The study found that 36.7% of fathers exhibited good involvement in childcare practices. While a majority contributed financially (55.6%) and engaged in play (55.4%), significantly fewer fathers participated in essential caregiving activities like feeding (27.3%) or seeking healthcare during illness (17.1%). Multivariable analysis identified significant predictors of paternal involvement: completing secondary education (AOR = 5.24, 95% CI: 2.64–10.38), having male children (AOR = 1.69, 95% CI: 1.094–2.62), first birth order (AOR = 5.66, 95% CI: 2.79–11.45), and fewer family size (AOR = 4.82, 95% CI: 2.58–9.016).

**Conclusion:**

This study underscores the limited yet essential role of fathers in child care within the Toke Kutaye District, revealing a need for targeted interventions to enhance paternal engagement. Promoting educational initiatives could serve as a strategy to foster deeper paternal participation in child-rearing practices, ultimately contributing to improved health and developmental outcomes for children.

## Introduction

Male involvement in child care is increasingly recognized as a vital aspect of fostering positive outcomes for children, particularly in supporting their health and development. This includes the investment of time and attention and the important role that a father usually plays in ensuring access to all essential resources such as nutrition, health services, and education (1, 2). It is the earliest period in a child’s life, namely the first 2 years when fathers’ involvement profoundly impacts children’s physical and emotional health (2).

Fathers are believed to be the final decision-makers in family matters, which include budgeting, allocating resources, and managing health services. This is also supported by the fact that caring fathers’ enrichment to children’s cognitive and social development is reflected in better academic performance and emotional stability (3, 4). There is evidence that children of involved fathers have fewer risk factors for behavioral and psychological problems and have greater predictability and security due to the two-parent care model (5). However, despite these benefits, the focus of research and interventions has predominantly focused on mothers, often neglecting the importance of paternal involvement in child-rearing (6).

In Ethiopia, as in many other developing countries, the impact of low male involvement in childcare is more evident. There is a significant gap in public awareness about the benefits of responsive care, and many children are therefore deprived of the caring relationships that support their development (7). Mortality rates among newborns and children under 5 years of age remain high in the region, with Ethiopia ranking fourth in the world in neonatal mortality. Therefore, there is an urgent need to address these public health concerns (4, 8). Interventions that focus on improving child health are consistent with the SDGs, as WHO supports the inclusion of men as one of the important strategies to improve health outcomes (9).

The role expectations of fathers in raising their children are partly determined by deeply rooted cultural norms. In many societies, fathers are conventionally viewed as breadwinners, while the responsibility for caregiving undoubtedly falls on the shoulders of mothers. Segmentation tends to result in fathers being under-involved in areas such as child nutrition and health care (10, 11). For example, studies in sub-Saharan Africa have documented that fathers who do not engage in nutrition practices are most likely to place their children at high risk of malnutrition and expose themselves to serious health complications (12, 13). In Nigeria, research suggests that about 57% of husbands actively engage in their children’s health care, highlighting the possibility of increased paternal involvement in different regional settings (14).

In addition, fathers tend to view their role in childcare primarily as a financial contribution rather than as an active involvement in childcare (15). The income difference between parents is also a very important socioeconomic variable that affects a father’s involvement. Studies have shown that when men earn significantly more than their partners, they tend to be less involved in day-to-day child care (16). However, studies also show that the higher the level of education, the more involved fathers are, suggesting that educational programs could be a key driver of changing attitudes and practices in child care (17). Family factors also influence fathers’ involvement in child care. Since only 33% of fathers in intact families report little or no active involvement, the mother’s employment and the quality of the relationship between the couple are modifying factors that increase the likelihood of father involvement (17). Other child-related factors include birth order and gender, with fathers reported to be more protective of firstborns and men (8, 18).

Father’s involvement in raising children has a major impact on health outcomes. However, despite growing recognition of the importance of nurturing and responsive relationships even in the early months, there are still significant barriers, including social norms and a lack of understanding by fathers of their important role. This finding highlights the need for targeted efforts to develop relationships between fathers and their children and also to involve fathers in childcare activities to enable the future development of healthier and better-adjusted children (4, 8). This dynamic also applies to developing comprehensive approaches to engage fathers in efforts that improve the health of their children and realize the full developmental potential of millions of children worldwide. Hence, it is essential to assess the father’s involvement in child care for planning an effective intervention strategy to improve male involvement (13, 19).

There is limited evidence on the proportion of male involvement and associated factors in child care in Ethiopia, particularly in the current study area. Therefore, this study aimed to assess the prevalence and associated factors of male involvement in child care among fathers of children under 2 years in Toke Kutaye district, Central Ethiopia, in 2024.

## Methods

### Study design, area, and period

A community-based cross-sectional study was conducted from January 28, 2024, to February 16, 2024, in Toke Kutaye District, which is 126 km from the capital, Addis Ababa and 12 km from Ambo town. This district has a population of approximately 140,422 and contains four health centers, 27 health posts, and one district hospital. Around 3,585 fathers with children under 2 years reside in the district.

### Population

The source population included all males with children under 2 years of age in the Toke Kutaye District during the study period. The study population consisted of randomly selected males with children under 2 years who met the inclusion criteria during the data collection period. The study unit was male individuals.

Inclusion criteria encompassed all married males with at least one child under 2 years residing in Toke Kutaye District for at least 6 months. Males not permanently residing in the study area and males who do not live with their wives or who are single.

### Sample size determination and sampling procedure

To determine the sample size, calculations were made based on the intended outcomes and previous studies. The sample size was calculated by using a single population proportion formula considering; Z = 1.96 with a 95% confidence level, 5% margin of error between the sample and population assumed, 39.2% the proportion of males involved in child care in the previous study conducted in Arsi Negele district, Ethiopia (18). The final calculated sample size was 403 after accounting 10% non-response rate. After applying a design effect of 1.5, the total sample size was found to be 403 × 1.5 = 605.

Simple random sampling was utilized across 50% (13 out of 27) of sub-districts. A list of eligible households was accessed through the Community Health Management Information System (CHMIS), and participants were randomly selected from this list for home interviews with assistance from health extension workers.

### Variables

#### Dependent variable

The dependent variable was male involvement in child care.

#### Independent variables

The independent variables investigated included socioeconomic and personal factors such as the father’s age, educational attainment, occupation, marital status, employment status, and household income. Family characteristics encompassed the mother’s age and educational background, the occupational status of both parents, as well as religion, ethnicity, and family size. Child characteristics included birth order, age, sex, and the total number of children in the family.

### Operational definitions

#### Male involvement in child care

According to this study male involvement in child care means the Participation of males in home-based activities regarding child care like feeding, diapering, changing clothes, playing, holding, bathing, and taking the child to health facilities for immunizations (35).

#### Measurements

Male involvement in childcare practice was assessed using 10-item questions. For each item, those who responded” yes” scored 1 and those who responded “No” scored 0. Based on the summative score from these 10 questions directed to the fathers, variable scores of 5 and above were considered good fathers’ involvement while variable scores of below 5 were considered poor fathers’ involvement in childcare practices (35).

### Data collection tools and techniques

The data collection tool was adapted and contextualized based on a literature review (5, 8, 18, 20) and underwent translation and back-translation from English to Afaan Oromoo (the local language) to ensure accuracy and consistency. The questionnaire comprised socio-demographic characteristics and questions evaluating male involvement in child care. Interviews were conducted in the fathers’ homes. Data collection proceeded after orally explaining the study’s purpose and benefits and obtaining verbal consent from each participant. A team of qualified health professionals collected the data and was supervised by investigators.

### Data quality control

A pre-test was done on 5% of the sample size before the actual data collection, and necessary adjustments were made based on the findings. To ensure a common understanding, data collectors were trained about the study’s aim, the contents of the tool, and data collection techniques. The investigators have supervised data collection daily to ensure its completeness and accuracy.

### Data processing and analysis

The data were checked, coded, and entered into Epi-data 3.1, then exported to SPSS 27 for cleaning and analysis. The data was checked for missed values and outliers, and descriptive statistics were calculated. Multicollinearity between independent variables was assessed using variance inflation factors. The Hosmer-Lemeshow test assessed model fit, with the final model fitting at a *p*-value of 0.27. Binary logistic regression was used to assess associations between dependent and independent variables. Variables with a *p*-value <0.25 were entered into multivariable analysis to determine independent factors. A *p* < 0.05 indicated statistical significance, and odds ratios were calculated for predictors of male involvement in child care.

## Results

### Socio-demographic characteristics of the participants

In this study, among 605 participants, 597 participants were interviewed and the response rate was 98.67%. The age of the participants ranges from 18 to 64 years with a mean age of 36.21 years with a standard deviation of (SD) 8.0268. Most (63.7%) of the study participants were Protestant by religion. Regarding occupation, 487 (81.6%) were farmers and (49.1%) completed primary school, and 22.9% completed secondary education (Table 1).

**Table 1:** Socio-demographic characteristics of the participants in the study “assessing male involvement in childcare and associated factors among fathers of below two in Toke Kutaye District, Central Ethiopia, 2024” (*n* = 597).

Variables	Category	Frequency	Per cent
Age of respondent (in years)	18–24	26	4.5
	25–29	89	14.9
	30–34	148	24.6
	35–39	157	26.3
	40–44	93	15.6
	45 and above	84	14.1
The marital status of the respondent	Married	594	99.5
	Widowed	3	0.5
Religion of respondent	Protestant	380	63.7
	Orthodox	209	35
	Wakefata	8	1.3
Ethnicity	Oromo	580	97.2
	Amara	16	2.7
Educational status of the respondent	No formal education	148	24.8
	Primary Education (1–8)	293	49.1
	Secondary Education (9–12)	137	22.9
	Diploma and above	19	3.2
Occupational status of the respondent	Farmer	487	81.6
	Merchant	32	5.4
	Government employee	11	1.8
	Daily laborer	39	6.5
	student	28	4.7
Age of child in a month	<12 months	253	42.4
	13–24 months	344	57.6
Sex of child	Male	319	53.4
	Female	278	46.6
Number of families living together	<5	354	59.3
	6 and above	243	40.7
Average monthly income	<1,000	95	15.9
	1,001–2,500	229	38.4
	2,501 and 5,000	164	27.5
	5,000 and above	109	18.3

### Level of male involvement in child care

**Figure 1:**
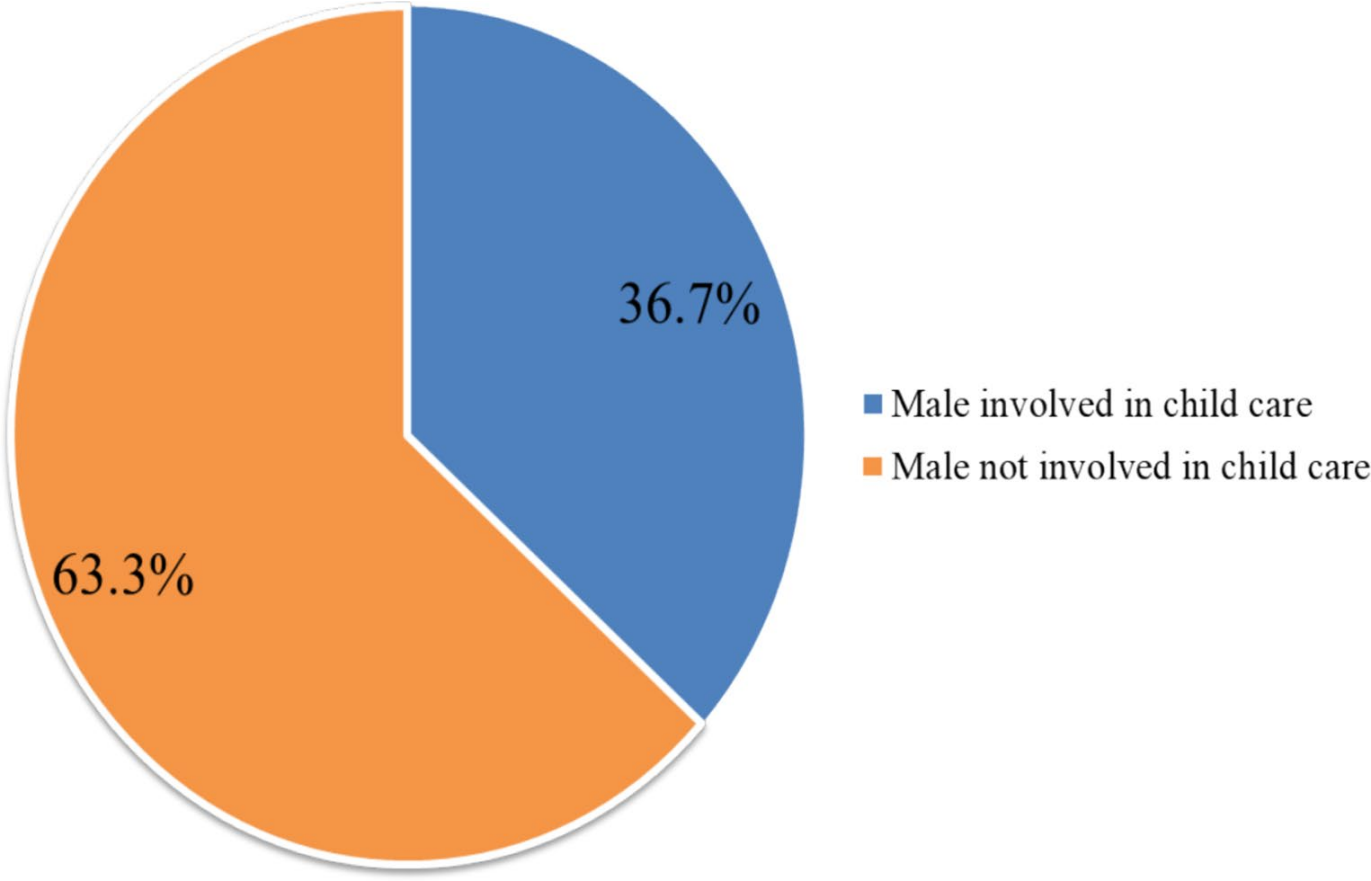
Percentage distribution of overall involvement of males in child care in Toke Kutaye District, Central Ethiopia, 2024.

The prevalence of good male involvement in child care among fathers with children under 2 years of age was 36.7% (95% CI: 32.8, 40.7) (Figure 1).

Most males, 332 (55.6%), contributed financially to support the mother, while 331 (55.4%) engaged in handling or playing with the child. Approximately one-quarter of the fathers, 163 (27.3%), participated in feeding their child, and 102 (17.1%) took the child to a clinic or health facility during illness in the past month. The list activity males involved was child diapering in which only 46 (7.7%) actively participated in changing diapers for the child (Table 2).

**Table 2:** Types of activities fathers participated in the study “assessing male involvement in childcare and associated factors among fathers of below two in Toke Kutaye District, Central Ethiopia, 2024” (*n* = 597).

Variables	Frequency and percentage	
	Yes (*N*/%)	No (*N*/%)
Financial support to the mother	332 (55.6)	265 (44.4)
Handling or playing with a child	331 (55.4)	266 (44.6)
Feeding the child	163 (27.3)	434 (72.7)
Taking the child to Health Facilities during illness	102 (17.1)	495 (82.9)
Taking the child to Health Facilities for Immunizations	51 (8.5)	546 (91.5)
Provide money to buy food for the child	328 (54.9)	269 (45.1)
Changing cloth	206 (34.5)	391 (65.5)
Participation in the childcare plan	314 (52.6)	283 (47.4)
Child bathing	93 (15.6)	504 (84.4)
Child diapering	46 (7.7)	551 (92.3)

### Factors associated with male involvement in childcare

In this study, all independent variables were assessed for their association with the outcome variable using Crude Odds Ratios (COR). The bi-variable analysis revealed that the educational status of the respondent, the mother’s age, the mother’s educational status, the child’s birth order, the child’s sex, and family size were significantly associated with male involvement in child care, with a *p* < 0.25. These variables were subsequently included in the multivariable regression model.

After adjusting for confounding variables using multivariable logistic regression, the educational status of the respondent, the sex of the child, birth order, and family size remained significantly associated with male involvement in child care. Specifically, males who attained secondary education were 5.24 times more likely to exhibit good involvement in child care compared to those who had not received formal education (AOR = 5.24, 95% CI: 2.64, 10.38). Fathers of male children were 1.69 times more likely to be involved in child care than fathers of female children (AOR = 1.69, 95% CI: 1.094, 2.62). Fathers whose child’s birth order was first were 5.66 times more likely to demonstrate good involvement in child care compared to those whose child was not the firstborn (AOR = 5.66, 95% CI: 2.79, 11.45). Additionally, males from families with five members or fewer were 4.82 times more likely to be involved in child care than those from families with more than six members (AOR = 4.82, 95% CI: 2.58, 9.016) (Table 3).

**Table 3:** Bi-variable and multivariable analysis of factors associated in the study “assessing male involvement in child care and associated factors among fathers of below two in Toke Kutaye District, Central, Ethiopia, 2024 (*n* = 597).

Variables	Category	Male involvement		COR (95% CI)	AOR (95% CI)
		Yes	No		
Respondent educational status	No formal education	23	1	1	1
	Primary education (1–8)	101	2.859 (1.72, 4.741) *	1.359 (0.731, 2.527)	1.359 (0.731, 2.527)
	Secondary education (9–12)	88	9.760 (5.54, 17.18)*	5.24 (2.64, 10.38)**	5.24 (2.64, 10.38)**
	Diploma and above	7	3.17 (1.292, 8.905)	1.527 (0.400, 5.833)	1.527 (0.400, 5.833)
Age of mother	18–25	92	5.160 (3.01, 8.823)*	0.580 (0.259, 1.302)	0.580 (0.259, 1.302)
	26–35	100	2.279 (1.37, 3.776) *	0.886 (0.443, 1.773)	0.886 (0.443, 1.773)
	36 and above	24	1	1	1
Mothers’ educational status	No formal education	63	1	1	1
	Primary education (1–8)	143	0.294 (0.128, 0.676) *	1.556 (0.977, 2.479)	1.556 (0.977, 2.479)
	Secondary education (9–12)	13	0.786 (0.348, 1.776)	1.894 (0.664, 5.405)	1.894 (0.664, 5.405)
Sex of the child	Male	147	2.445 (1.72, 3.46) *	1.695 (1.094, 2.62)**	1.695 (1.094, 2.62)**
	Female	72	1	1	1
Birth order	First	121	17.71 (10.61, 29.5) *	5.66 (2.79, 11.45)**	5.66 (2.79, 11.45)**
	Last	36	1	1	1
Family size	<5	190	8.549 (5.50, 13.28) *	4.82 (2.58, 9.016)**	4.82 (2.58, 9.016)**
	6 and above	29	1	1	1

*Significant variables at *P* < 0.25 in the bivariate analysis. **Statistically significant variables at *p* < 0.05 in the multivariable analysis. COR, Crude odds ratio; AOR, Adjusted odds ratio; CI, Confidence interval; 1: reference category.

## Discussion

Fathers play more responsibilities in the care of infants and young children, due to the new social changes that accept the involvement of fathers. Although there exists tremendous social recognition of the bond between mother and infant, a period in infancy has equal implications for fathers’ involvement in caregiving. Nonetheless, this is a fairly unappreciated topic of today’s analysis.

However, our study revealed only 36.7% of father’s practice a good level of childcare involvement and this is almost similar to the 39.2% of fathers reported from the rural Arsi Negele district of Oromia, Ethiopia (18). This might be attributed to the fact that on average, child care is primarily a mother’s responsibility, while fathering is seen more as a side option. In addition, because of their obligations to their workplace, many fathers are often away from home and, therefore, deny themselves the opportunity to care for their children. This is also true from the results obtained in a study conducted among a community in an urban slum in Bangalore, India where father involvement was portrayed as differing according to cultural context (15). Paternal involvement in childcare and related activities across different regions of the world has been measured variously and the level of engagement varies according to local circumstances including the cultural environment (21–23).

Nearly half of the fathers, 332 (55.6%) said that they are supporting their families financially; this has shown that fathers are also responsible for the welfare of the family even though they may not be involved much in taking care of the household responsibilities. This is in line with other research that suggested that men tend to focus on financial provision and therefore see themselves more as breadwinners than caregivers regarding childcare issues (4, 15, 24, 25). Although 331 fathers (55.4%) played with their children, an important activity that helps to strengthen the affectionate tie with the child, only 163 fathers (27.3%) fed their children, and only 102 fathers (17.1%) sought care for their sick children in the last 1 month. These results seem to mirror patterns in more minimal involvement in essential caregiver roles; the persistent sex roles make it even more challenging for men to participate in direct childcare responsibilities. For example, the lack of interest in diaper changing was indicated by only 46 fathers (7.7%) This shows that there are still lingering cultural practices that consider more traditional attachment to fathering as inferior to that of mothering.

### Factors affecting male involvement in childcare

The current study found that fathers with male children are highly likely to be involved in childcare more so actively. This is contrary to studies conducted in South India that showed fathers of boy children to be less involved than fathers with girl children (17). There may be a cultural influence in our study area where the birth of a male child is festered more joyously resulting in fathers being more involved. For instance, one of the cross-sectional descriptive studies conducted at Gemza Woreda, Ethiopia showed that fathers with male children had 3.97 odds of practicing paternal childcare than fathers with female children (26).

In addition, the first birth order of the child was statistically significant and found to be directly related to paternal involvement which was supported by studies conducted in southern India suggesting that first-time fathers are more involved than those with subsequent children (17). This might be due to the first-time parents’ passion and enthusiasm to address the needs of their children.

Another demographic characteristic that was found to have influenced the level of involvement of fathers in childcare was their education level. Fathers with secondary-level education were also more likely to engage fully in childcare activities, as has also been observed in Nepal where access to education is associated with the active promotion of breastfeeding and other child care practices (27). This may be explained by heightened awareness concerning the father’s engagement in child development hence active participation in caregiving roles (28).

Family size has also shown a significant association with the good involvement of fathers in child care. Fathers from fewer members of a family size had higher involvement in providing care for their children when compared to those from large family sizes. This finding is supported by a study done in Ethiopia in which fathers’ involvement in child care is less among larger family size (29).

### Implications of the findings

The study on the involvement of fathers with children under 2 years of age in Toke Kutaye district, Ethiopia, found that only 36.7% of fathers participate significantly in important care activities, suggesting a critical gap in parent involvement. Key factors that influence this participation include the child’s level of education, gender and birth order, and family size. These findings suggest the need for targeted interventions, such as educational initiatives to raise awareness of active paternity and community campaigns to challenge traditional gender norms. By promoting cooperative parenting and incorporating paternal involvement into maternal and child health strategies, these efforts can contribute to better health outcomes for young children in the region and help reduce neonatal and child mortality rates among children under 5 years of age.

### Strengths and limitations of the study

This study’s strengths include its large sample size of 605 fathers, enabling robust statistical analyses and reliable findings on male involvement in child care in Toke Kutaye, Ethiopia. The use of a structured questionnaire facilitates accurate data collection on various socio-demographic factors associated with paternal involvement. However, limitations include potential biases in self-reported measures, which can lead to overestimation of involvement levels. Additionally, cultural norms may influence responses, and the cross-sectional design restricts causal inferences. Finally, data collection occurred within a specific time frame, which may not reflect seasonal variances in parenting behaviors.

## Conclusion

This study highlights fathers’ critical, yet often underappreciated, role in child care within Toke Kutaye District, Ethiopia. With only 36.7% of fathers demonstrating good involvement in essential caregiving practices, there is a clear gap that needs to be addressed. The findings suggest that educational attainment, the sex of the child, birth order, and family size significantly influence paternal engagement in child-rearing. To improve child health outcomes, targeted interventions aimed at increasing male involvement in feeding, health care, and nurturing activities are essential. Moreover, community awareness programs should challenge traditional gender roles that limit paternal participation and promote a more collaborative approach to parenting. By fostering a culture that values and supports active fatherhood, we can enhance child development and overall family wellbeing, ultimately contributing to better health outcomes for young children in the region.

## Data Availability

The original contributions presented in the study are included in the article/supplementary material, further inquiries can be directed to the corresponding authors.
